# “We cannot do away with exams: Parents believe in them, so does the wider community”. Reimagining the examination system in the Maldives

**DOI:** 10.1007/s11125-022-09613-w

**Published:** 2022-09-01

**Authors:** Abdulla Sodiq, Rhonda Di Biase

**Affiliations:** 1 School of Education and Social Work, Birmingham City University, City South Campus, Birmingham, B15 3TN UK; 2 Melbourne Graduate School for Education, University of Melbourne, Melbourne, Australia

**Keywords:** Examination systems, Equity, Secondary schooling, Pedagogy, Curriculum, Small states

## Abstract

This article presents an exploratory analysis of the external secondary examination system in the Republic of Maldives. The school system is structured around primary grades following a local national curriculum, secondary grades leading to O-level (Ordinary Level) examinations and higher secondary grades leading to A-level (Advanced Level) examinations. Based on desk data, the article analyses different dimensions of secondary and higher secondary education enrolments and attainment levels. It considers the implications from the reliance on British international examinations for students and schools. In addition, there is an exploration of the National Curriculum and equity in secondary education in relation to gender-specific outcomes and outcomes for students in rural atolls in comparison to the outcomes in urban capital island, Male’. The article concludes by considering alternatives to the reliance on international examinations and potential options for national certification that may be more aligned to local needs and relevant to the context.

## Context for change

“We cannot do away with exams—parents believe in them, so does the wider community” (UNESCO, 2020a)

The entrenched examination systems found in many countries find widespread support from many stakeholders. However, what they are perceived to offer and what they deliver may be at odds. Further, according to Ball et al. (2012, p. 139), “the ways in which we are all deeply implicated in, and bound up and into, the contemporary neo-liberal and globalizing settlement…[means] that most of the time we do not even notice it is there”. The Covid-19 pandemic has disrupted education systems and challenged them to adapt. The World Bank, in response to the pandemic, asks whether “reinstituting the pre-Covid-19 exam system is the best course of action” (World Bank, 2020, p. 34). They conceptualize an opportunity to make education systems stronger and more inclusive than they were pre-Covid-19.

Writing in a special issue on education and the pandemic within this journal, d’Orville (2020, p. 13) wrote: “Countries should use the focus and innovativeness of the recovery period to ‘build back better’. The key is not to replicate the apparently vulnerable pre-Covid-19 systems, but instead to build improved systems”. The disruption of the pandemic takes place in the era of the Sustainable Development Goals (SDG) with their focus on providing an inclusive, equitable, and quality education for all learners (UNESCO, 2017). SDG4 puts increased focus on the provision and quality of secondary education. Stromquist (2020, p. 47), in the same special issue, argued, “Upper secondary school completion is a persistent challenge for low-income countries…the issue of cycle completion is important, for it affects students’ transition from primary to secondary schooling and from secondary schooling to college”.

Cairns (2020) refers to the pandemic as an opportunity to rethink the efficiency of high-stakes examinations. And in the same special issue on the impact of Covid-19 on education, Zhao (2020) argues that the Covid-19 situation should be treated as an opportunity for long-term change in education systems.

This paper presents an exploratory analysis of the secondary and higher secondary situation in the Maldives, a Small Island Developing State (SIDS). SIDS have unique challenges due to their geographical and demographic characteristics. Considering recent expansion of the availability of secondary schooling across the atolls, this paper presents insights into current enrollment and achievement rates for both secondary and higher secondary education (HSE), comparing the capital, Malé, to the atolls. We examine the enrollment and achievement opportunities across the country to document trends over time and consider equality of opportunity nationally and whether the current secondary examination system is fit for its purpose.

If Covid-19 does indeed present an opportunity to think innovatively and leverage off the disruption caused by the pandemic, we see this analysis of the current status quo as a first step in considering future possibilities in the Maldives. President Solih has publicly endorsed the necessity for Maldivian young people to develop the skills to play an active and pivotal role in helping the country rebuild post-pandemic and has announced a free HSE scheme, explicitly linking school success with post-school pathways. Considering the specific needs within SIDS and the critical need for education systems to help grow the country’s human resources, this analysis is timely. In this paper we draw on secondary data to better understand the current enrollment and achievement situation nationally and consider these findings in light of historical trends and earlier analyses of O- and A-level examinations.

## Maldives context—a small island developing state

**Figure 1:**
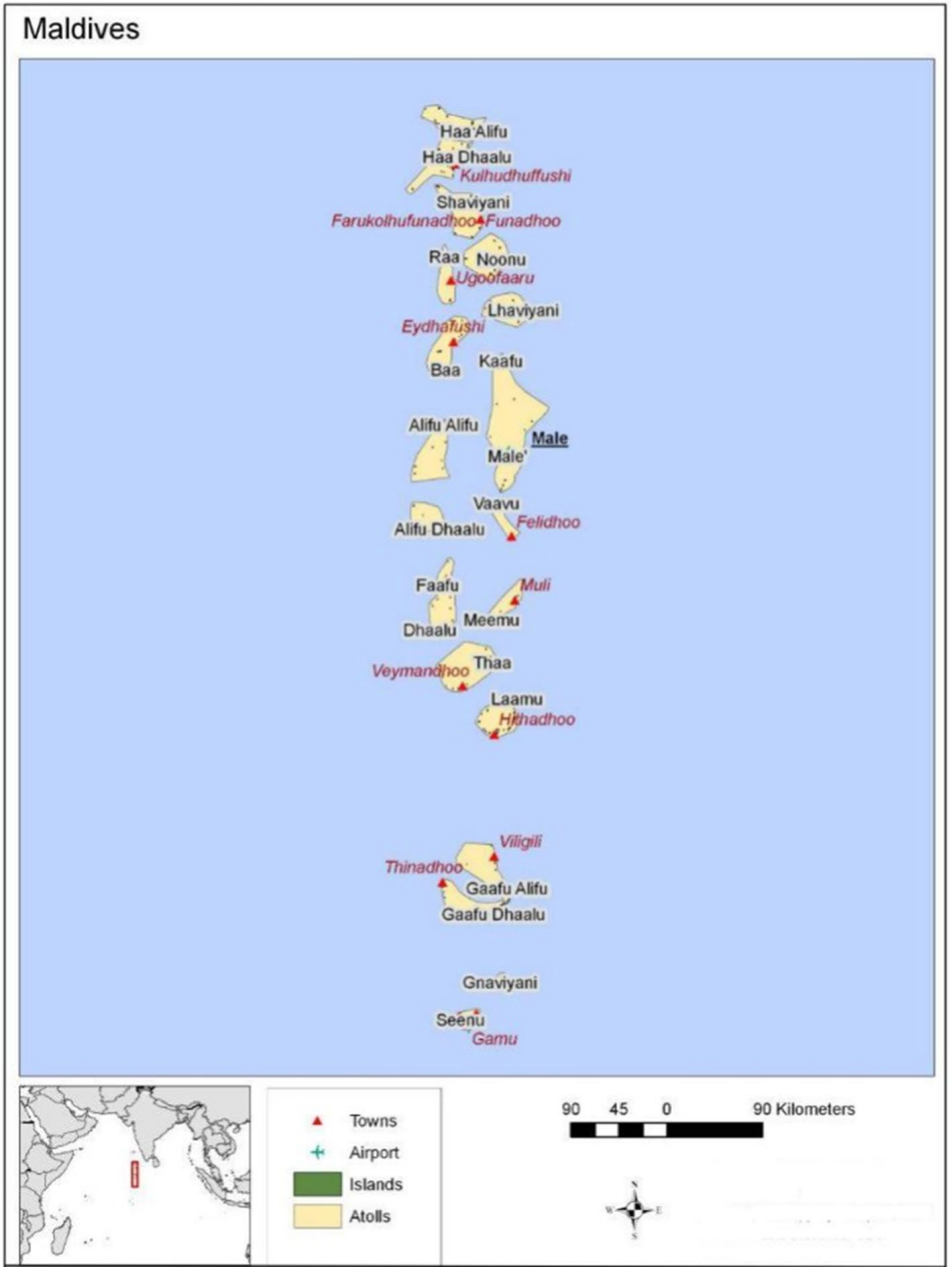
Map of Maldives *Source*: WHO, 2005

The Maldives, as a SIDS, consists of 188 inhabited islands spanning 1000 km from north to south. The country is geographically dispersed, with most Maldivians living on small islands in 26 naturally occurring atolls, divided into 20 administrative atolls (Figure 1). Malé, the capital, is densely populated, containing approximately a third of the population. Of the inhabited islands, 91 islands have populations of less than 1000, and only 4 islands have populations greater than 5000.

As a SIDS, the Maldives faces unique challenges. Apart from a geographically dispersed population, its small size means limited resources, and susceptibility to environmental challenges such as threats from climate change and sea-level rise. It has few resources that can drive economic activity, apart from tourism, which is vulnerable to external events, as was all too evident through the Covid-19 pandemic. Small states tend to look outward to exploit their narrow resource base (Bacchus, 2008). This reliance on tourism and the impact of any downturn in numbers can be seen not only in the current pandemic but following the 2004 Boxing Day tsunami. Both events have critically impacted tourist arrivals. With 25% of GDP reliant on tourism (World Bank, 2021), such incidents have devastating effects on the economy.

Tourism as the economy’s main driver has also exacerbated existing inequities. In her analysis of tourism development, Schevyens (2011) writes that income disparity between Malé and other atolls has increased, entrenching Malé as the development center and compounding the underdevelopment of outer atolls. There is greater development on atolls where resorts provide employment opportunities than on atolls with limited access to the benefits of tourism. The disparity is also reflected in schools with a higher level of resources, both human and material, such as Malé’s schools.

### Structure of the education system

**Figure 2:**
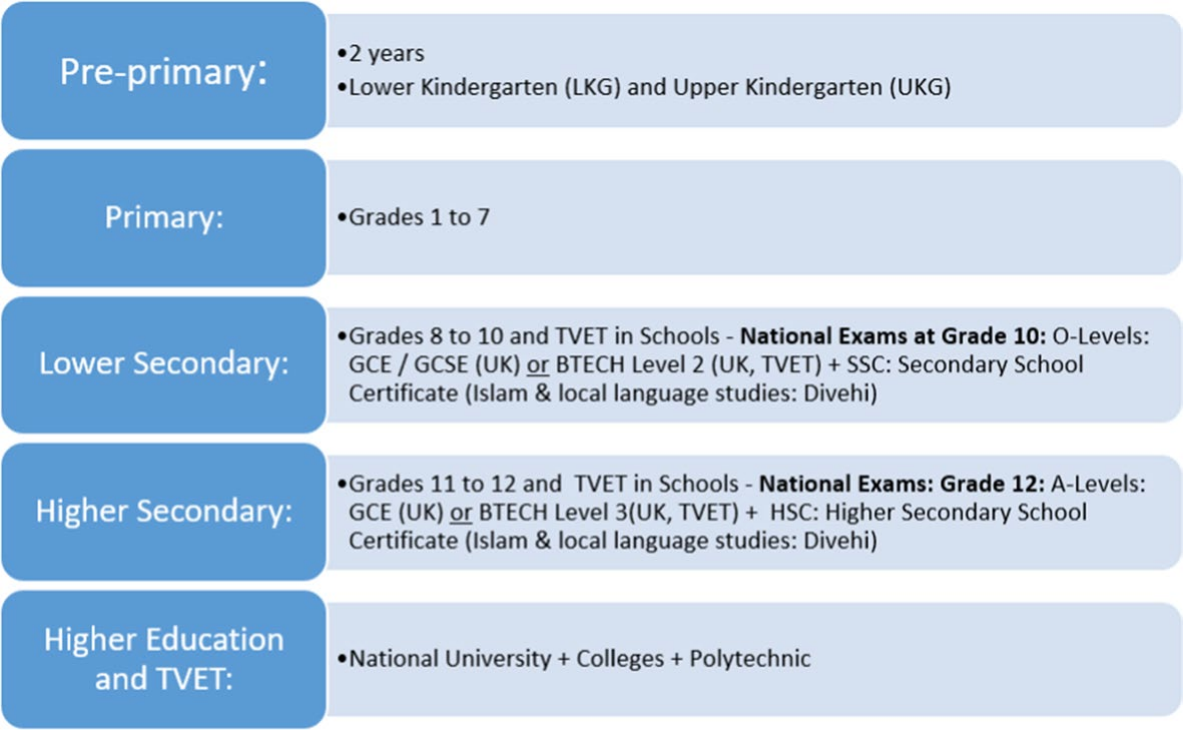
Maldives education system *Source*: Adapted from MoE, 2019

The school system is structured around primary grades (1–7) following a local national curriculum. Lower secondary students prepare for the British International General Certificate of Secondary Education (IGCSE) and General Certificate of Education (GCE) examinations (GCE O level) in six subjects, including two local subjects (Islam and Dhivehi). At the end of HSE, students sit the General Certificate of Education Advanced Level examination (GCE A-Levels) administered by Edexcel, UK (Figure 2).

**Figure 3:**
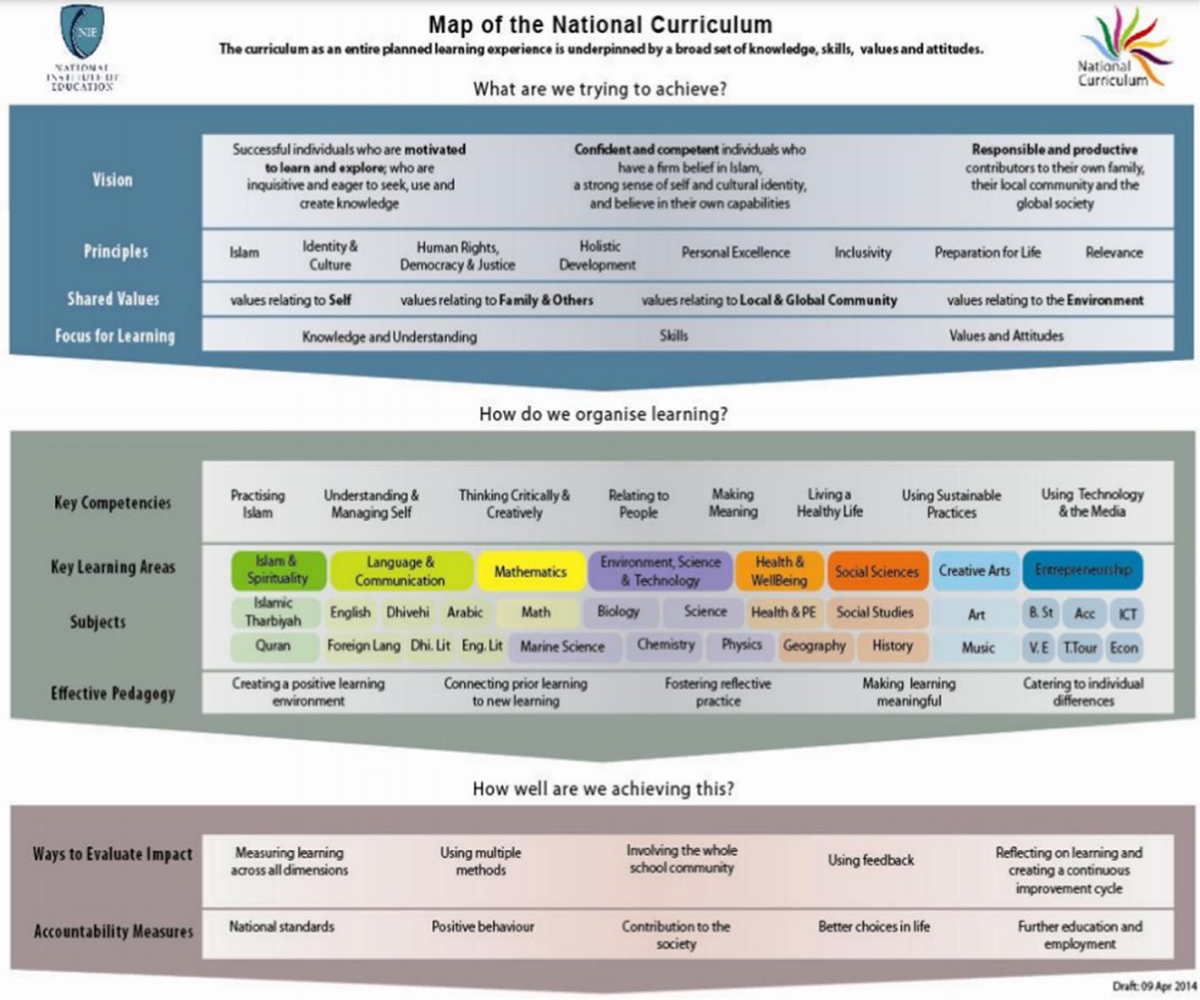
Outline of the National Curriculum Framework *Source*: National Institute of Education, 2015

In 2015 a new National Curriculum Framework (NCF), covering grades 1–10, was a major reform. The NCF is an outcomes-based curriculum structured around eight key competencies and key learning areas (Figure 3). It promotes a more holistic approach and a definite shift from the preceding, more prescriptive syllabus that outlined, through schemes of work, what should be taught each week. The NCF clearly articulates pedagogical dimensions, which together with the key competencies put focus on not just what should be taught but how it should be taught.

From 2014 diversified options have been offered at the secondary level. The Business and Technology Council (BTEC) program from the UK began in 2014 as a new secondary stream. Of 212 government schools in 2017, 181 schools offered this BTEC stream. Students begin in Grade 8 and study one BTEC Level 2 course. The BTEC stream continues as an option at upper secondary as BTEC Level 3 courses. Another local program, Dhasvaaru, was introduced in 2014 as an alternative pathway in lower secondary grades. Starting with 11 schools in 2014, Dhasvaaru expanded across the country (Di Biase & Maniku, 2021), recognizing the need for programs designed to cater to the diversity of students’ skills and aspirations.

### History of O- and A-level exams

The development and administration of examinations requires a high level of expertise. Small states, with their limited human resources, have found different ways to manage their examinations, including local, regional, and external examinations (Bray & Adam, 2001). The Maldives adopted external examinations for English medium schools in Malé in 1984, designed around the Edexcel O-level and A-level examinations. Essentially, then, a dual system was in place—traditional Dhivehi schools in the atolls and English medium schools in Malé. Until 1992, secondary education was only available in Malé. What is unique to the Maldives is that international examinations from the UK are used at a system-wide level as secondary certification (Yamada et al. 2015). It is not, as in some countries, a choice to sit the international O-level exams rather than a local national certificate. Yet, as Yamada et al. (2015, p. 61) ask, “Does the national adoption of an international certification equally affect students who take the exams?” This question is a product of how the use of O-level examinations has evolved over time as the education system expanded. Consequently the question is very relevant. Bray and Adam (2001) analyzed the various complexities around this issue for small states. They revealed that 77% of students failed the English examination in 1999. This speaks to the large numbers of students who, over the years, have experienced failure in sitting these exams (World Bank, 2012; Yamada et al., 2015). Moreover, the English medium of instruction further differentiates Malé parents, who tend to have higher levels of English fluency, from island populations, positioning some families better than others for these examinations (Yamada et al., 2015).

Bray and Adam (2001) raised other shortcomings of the external examination system: the cost (relying on UK materials); the nature of the curriculum related to subject offerings; bias in questions, which also assumes the fluency of a native English speaker; and the elitist nature of the qualification. Further, Ali (2018), reporting on this era, noted that students in atoll schools were often confined to subject options such as commerce and business, as science subjects required school laboratories and resources that were not available in the atolls. It can also be noted that, when based on external examinations, the education system does not reflect the everyday lives of Maldivian students, resulting in serious damage to the equity of education (Bray & Adam, 2001).

Yet, the O-level examinations remain a pillar of the education system in the country. While enrollment in secondary grades has increased, Yamada et al. (2015) raised concerns around students’ experience of schooling, the overall pass rates, and the pedagogical effects of focusing on results. Importantly, they contend that while the O-level exams are designed to serve the masses, they remain biased toward elites, with socioeconomic and home environment factors playing a key role in a student’s success. They also highlight that more experienced teachers tend to be found in Malé, exacerbating disparities between Malé and the atolls*.*

As a SIDS, the Maldives’s demographic and geographic conditions pose a unique set of challenges for effective and efficient provision of education across the country. Yamada et al. (2015) point to difficulties in managing education across the atolls in an efficient manner, also noting it is challenging to develop curricula, monitoring mechanisms, and teacher education programs. They conclude that the British examination system is a realistic strategy for managing the national system, also noting parents prefer the international certification. Yet they also point to the obvious tensions in the system: particularly, the Ministry of Education (MoE) emphasizes improving O-level results, which may run counter to the pedagogical vision embedded within the NCF. They contend that the pedagogical aims of the NCF to encourage learner-centered pedagogical approaches do not align with efforts to improve O-level examination results. As Shareef (2016) writes:

…a lot of pressure is imposed on the schools to prepare students for the external examination. …this trend has a direct impact on the teaching methodology used by teachers. Teachers regard their prime duty as being to transmit effectively a body of knowledge to children. (p. 142)

Hence, a lot of pressure is imposed on Maldivian schools to prepare students for these external examinations, impacting on the teaching approaches used by teachers.

### Examination systems and Covid-19

During this pandemic and its disruption to school and examination systems, the nature and role of examinations has also been called into question:

… After students return to school, countries should ask themselves whether simply reinstituting the pre-Covid-19 exam system is the best course of action...focusing of curriculum and greater attention to student well-being may spur moves to reform exam systems that are currently viewed as undermining real learning. (World Bank, 2020, p. 34)

This is an interesting viewpoint, given the tension identified with focusing on exam results in the Maldives versus the vision for holistic learning embedded in the NCF, in particular the pedagogical dimensions.

A related perspective can be seen in Cairns’s (2020) analysis of the examination system in Victoria, Australia, after a lengthy school shutdown in 2020 that continued up to the final exam period. She studies the relationship between high-stakes examinations and how the curriculum is enacted and finds that demands of accountability and performance strongly influence teaching approaches. She also outlines how examinations have been associated with equity and meritocracy, seen as an impartial means of assessment and proposed as a way of providing education opportunities to all. Importantly, she also points to issues related to equity arising from the emphasis on performance, leading to a growing disparity between students and schools in terms of the resources connected to the performative paradigm. Further, she contends that issues around equity and access have become more acute during Covid-19. Such analysis is relevant to the Maldives, given the disparity of human and material resources between Malé and atoll schools.

### Moving forward

Serious inequalities exist in the Maldives in spite of its graduation to a middle-income country in 2013. In 2014, students in Malé were found to complete three additional years of schooling compared to students in atoll schools (UNDP, 2014). The provision of quality, widespread education is particularly important for small island states, given the need to prepare students for further post-schooling options, both higher education and employment (Latheef & Gupta, 2007). Consequently, education systems in small states play a core role in building human resource capacity (Bacchus, 2008), now critically important in how the Maldives can confront the challenges arising from Covid-19 (The President’s Office, 2020). Understanding the outcomes of the O- and A-level exams across the country is important in considering equity of opportunity within the education system. The expansion of secondary education across atolls was intended to provide greater opportunities. However, to what extent does it serve this purpose? Hence the focus of this paper is to explore whether the availability of O-level education across the country promotes equity and A-level attainment.

## Research methods

The exploratory study presents an analysis of the participation and performance in secondary education and HSE in the Maldives. An examination of trends nationally—in the capital, Malé; and in the atolls—underpins this analysis. The research questions are:

1. What are the national and regional trends in enrollment and attainment patterns in the O- and A-level examinations?
2. What factors can be identified to explain these trends?
3. What are the implications of these trends for the equitable provision of secondary education nationally?

Our research relied on analyzing publicly available secondary and HSE data from the following data sets:

- Statistical Yearbook of the Maldives; National Bureau of Statistics, Department of National Planning and Infrastructure: 2013, 2018 and 2020Maldives Education Sector Analysis (ESA), February 2019, Ministry of Education.
- School Statistics 2019, Ministry of Education

We examined trends over time and identified performance patterns for various subgroups: students in Malé and the atolls, across atolls, and by gender. With SDG4 reporting requirements globally, there has been an emergence of more extensive publicly available data. In the past it has been difficult to access a wide range of robust data apart from student enrollment and teacher numbers.

## Results

In this section we present our analysis of trends identified from these data sources. We explore enrollment and more recently available gender-disaggregated and atoll-level attainment levels of secondary and HSE, and compare data in Malé with that from the atolls. We present the findings in graphs to illustrate trends, along with explanatory discussion of trends and outcomes.

### Overall O-level pass rates

**Figure 4:**
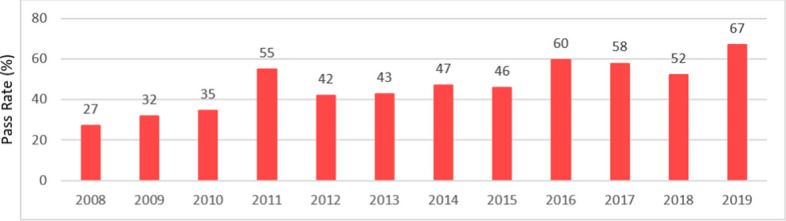
Pass rates (5 or more subjects) Data derived from: National Bureau of Statistics (2018, 2020, 2013)

O-level results up to 2015 are outlined in Figure 4, showing an average pass rate of 41% for the years 2008–2015. Pass rates ranged from 27% in 2008 to a high of 55% in 2011, which was not sustained over the years that followed. In 2016, there was an increase in the pass rate to 60% with results hovering over 50% in the subsequent years.

By 2019, pass rates had risen to 67%, reflecting the steady rise in GCE O-level achievement over the preceding years. Participation in alternative pathways, as illustrated in Table 1 and Figure 8 presented later in our findings, may help explain these results.

**Table 1:** Dhasvaaru program enrollment since 2014 Source: MoE (2019)

Year	Total	Cumulative total	Certified % since 2014
2014	151	151	n/a
2017	1364	3754	63%
2018	844	4598	74%

### Malé and atoll pass rates

**Figure 5:**
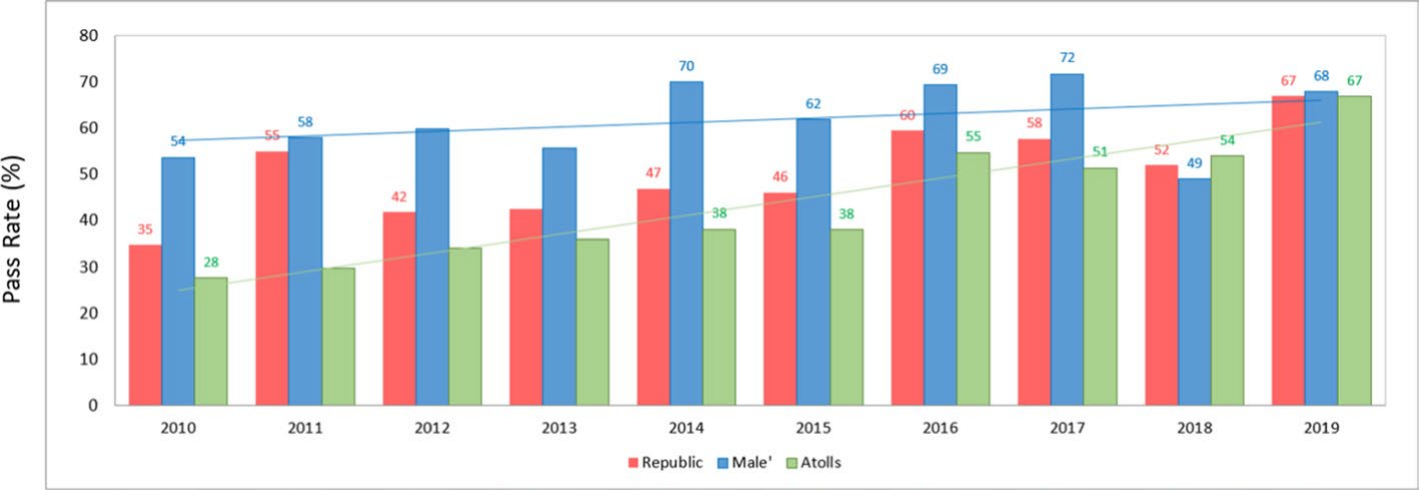
Pass % in GCE O-level comparing Malé and atolls *Source*: National Bureau of Statistics (2018, 2020, and 2013)

Figure 5 illustrates O-level pass rates comparing Malé with results in the atolls. Malé showed higher pass percentages until 2018, when the atolls outperformed Malé. In contrast, in 2010, the disparity between Malé and atoll schools was very apparent with pass percentage in Malé (54%) almost double the atoll percentage (28%). After 2017, there has been a clear narrowing of the disparity between the atolls and Malé, from a margin of about 20% in 2017 to a position of parity in 2019.

### O-level pass rate by gender

**Figure 6:**
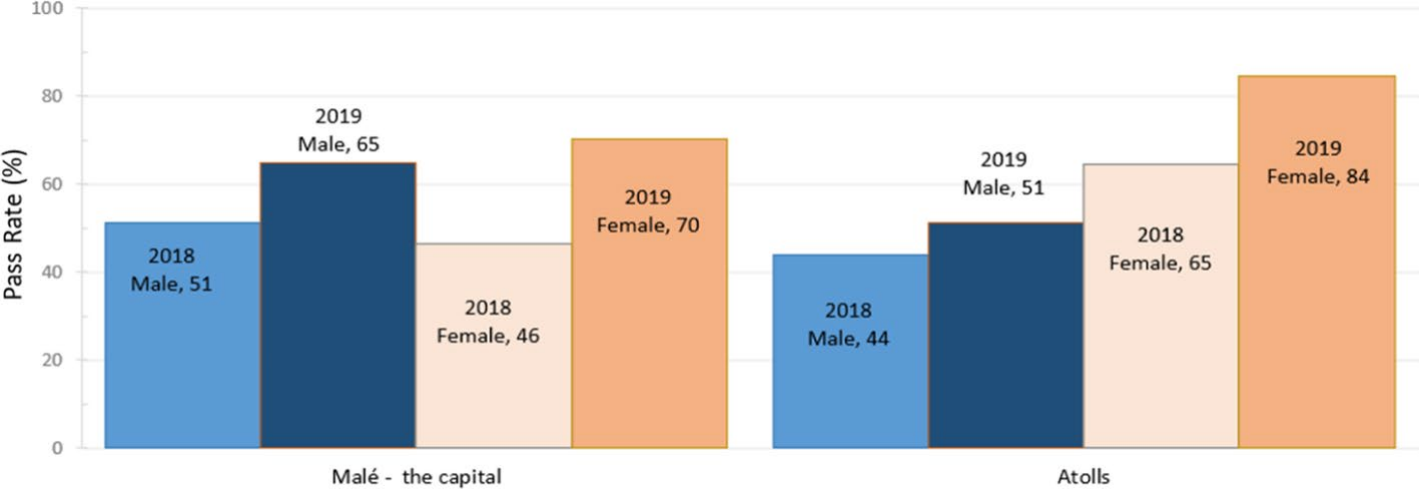
GCE O-level pass % by gender (2018–2019) *Source*: National Bureau of Statistics (2020)

Figure 6 shows GCE O-level pass percentages (Grades A–C in five or more subjects) disaggregated by gender. The strong performance of female students stands out in both Malé and in the atolls in 2019. Gains are markedly lower for boys in the atolls, from 44% to 51% (+7%) in the atolls between 2018 and 2019, compared to the gains for girls in the atolls, from 65% to 84% (+19%)—more than double the gain of the boys. The boys’ gain in the atolls is also relatively low compared to those in the capital (51% to 65%, gain of 14%). In other words, boys in the atolls have experienced lower gains in O-level passes than any other group*.*

### Atoll O-level pass rates

**Figure 7:**
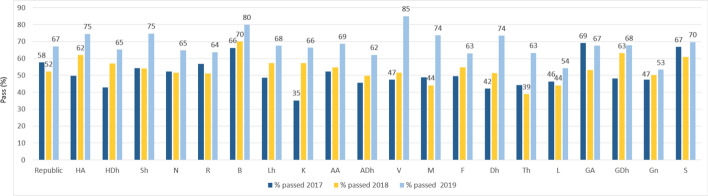
O-level pass rates (%) by atoll (2017–2019) *Source*: National Bureau of Statistics (2020)

There has been a steady increase in O-level performance in the atolls between 2017 and 2019 (Figure 7). The largest increase can be observed in Vaavu (V) atoll, from 47% pass rate in 2016 to 85% pass rate in 2019. Other atolls that demonstrated noticeable increases include Haa Alif (HA) atoll, with an increase from 50% to about 75%, well above the national level. Baa (B) and Seenu (S) atolls maintained high achievement rates throughout the period: Baa shifted from a 67% pass rate to about 80%, the second highest in the country. Lhaviyani (Lh) atoll, which was well below the national average at 48%, rose to 68%, above the national average (67%). Kaafu (K) atoll, which had the lowest pass rates in the country at 35%, increased to 66%, putting it on a par with the national average. Meemu (M), Dhaalu (Dh), and Thaa (Th) atolls demonstrated the highest gains in 2019 (74%, 74%, and 63%) after being amongst the lowest achieving atolls for the preceding two years. Finally, the five atolls Raa (R), Alif Dhaal (ADh), Thaa (Th), Laamu (L), and Gnaviyani (Gn) continued to underperform with O-level pass rates below the national averages over the three years. The only atolls consistently performing above the national average pass rates were Baa (B) and Seenu (S/Addu) Atoll. Overall, the national pass rate improved from 58% in 2017 to 67% in 2019.

### Participation in TVET (Technical Vocational Education and Training) programs

**Figure 8:**
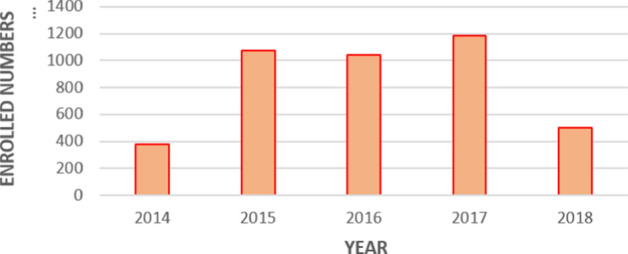
BTEC O-level program enrollment since 2014 *Source*: MoE (2019)

Between 2014 and 2018, the locally accredited Dhasvaaru program serviced over 4500 students (Table 1). Both Dhasvaaru and the international BTEC O-level program (Figure 8) may be seen as overall successes and attracted interest amongst the targeted students in the first 3 years. This coincided with the time when the decreasing national trends in O-level completion numbers stabilized slightly, especially in the atolls, between 2015 and 2017.

### Net enrollment ratios—Lower and secondary education

**Figure 9:**
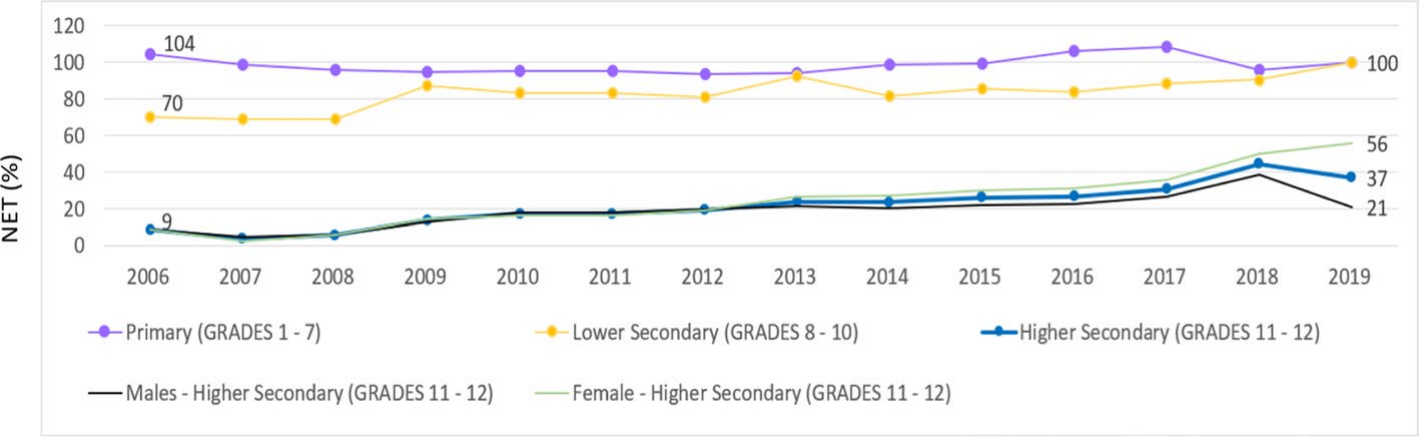
Net Enrollment Ratio (NER) by Level: 2006–2019 *Source*: National Bureau of Statistics (2020)

O-level results in lower secondary education may have an impact on engagement levels in HSE, where A-level exams are taken as the main qualification. Net Enrollment Ratio (NER) in HSE has been low but gradually improving between 2006 and 2019. It has stayed around 40% in recent years, compared to figures even lower, below 30%, in the years up to 2010 (Figure 9). Females have experienced the more significant improvement in NER, from a markedly low 9% in 2006 to 56% in 2019, while male pass rate reached only 21%. While access and completion of both primary education and lower secondary education reached 100% of the relevant population, HSE had remained worryingly low since 2006. Notably, there was a significant decrease in net enrollment for males in HSE, from about 40% in 2018 to 21% in 2019.

**Figure 10:**
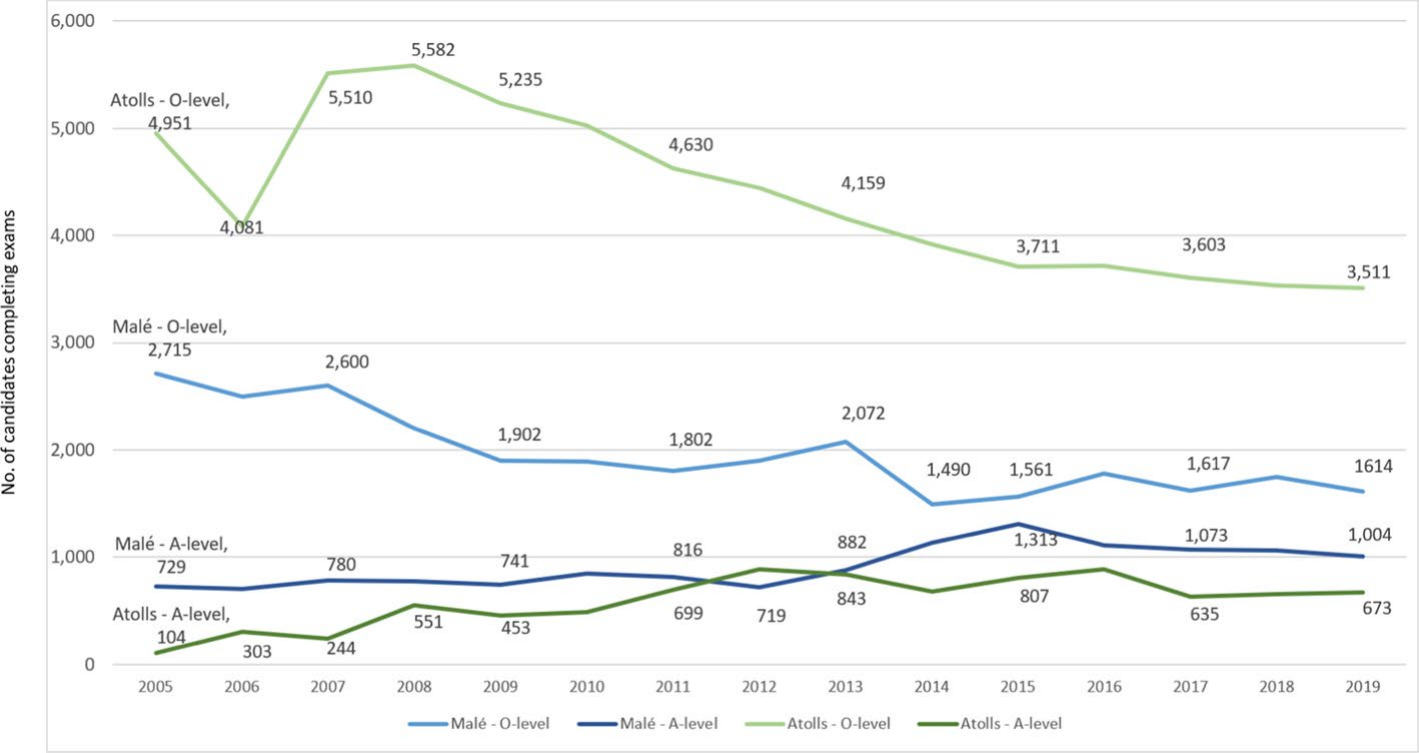
Trends in O-level & A-level examination completion numbers in atolls & Malé, 2005–2019 *Source*: National Bureau of Statistics (2020)

Both the atolls and the capital have seen a decrease in O-level exam entrance numbers since 2007, with a sharp decrease in the capital in 2014 from 2072 students to 1490 (Figure 10). The overall decrease in O-level completion numbers took place despite an improving NER for lower secondary education (Figure 9). This indicates that the decrease in actual O-level numbers is likely to be due to demographic changes in the population for this age group across the country.

There is a marked difference in student completion numbers between O level and A level for both the capital and the atolls, with the difference greater for the atoll student population. Since 2017, only a fifth of atoll students who completed O level sat the A-level examinations. For the capital, the difference has been smaller, just over a third of O-level entrance numbers completing A-level examinations since 2017 (Figure 10).

**Figure 11:**
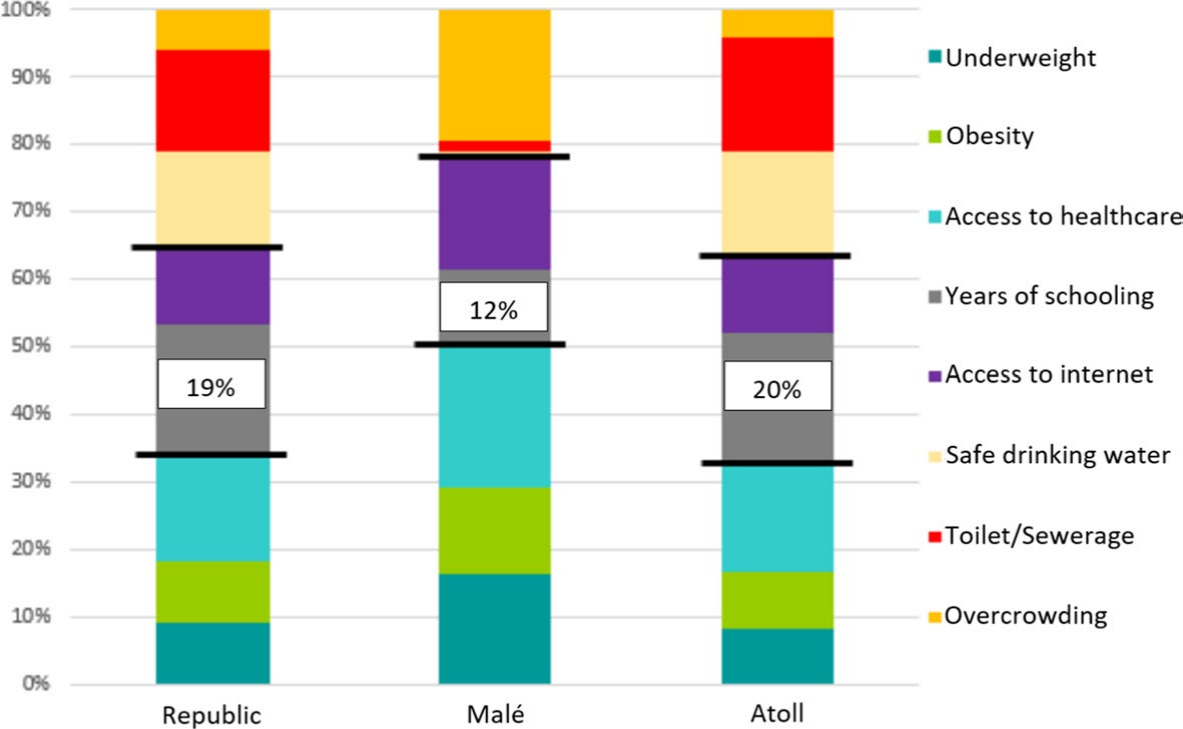
Multidimensional poverty indicators in the Maldives, 2016 From National Bureau of Statistics et al. (2020)

In order to understand potential effects of secondary education engagement on number of years of schooling, we analyzed data comparing the number of years of schooling and the associated effects between the atolls and the capital. According to the latest National Multidimensional Poverty Report (National Bureau of Statistics et al., 2020), years of schooling (less than 10 years of schooling for those aged 15+) is the main contributor to overall poverty in both the atolls and in the nation as a whole (20% and 19% respectively; Figure 11). In the capital, years of schooling have a lesser effect of Multidimensional Poverty Index (MPI) (12%).

Poor engagement in HSE education is reflected and confirmed in the low NER for HSE (Figure 9). This engagement level has been gradually improving over the years, however, which could also be a reflection of the significant increase in the number of schools offering upper secondary education across the country, especially in the atolls, where upper secondary school numbers increased from none in 2001 to 50 in 2019 (MoE, 2020; National Bureau of Statistics, 2020). Historically, such stark difference between O-level and A-level completion had been the greatest in the years prior to 2014, when O-level numbers gradually decreased for some 10 years both in the capital and the atolls (Figure 10). Since 2014, both O-level numbers have stabilized.

## Discussion

Our findings build on earlier analyses of access and attainment data related to the British O- and A-level exams since the 1990s (Bray & Adam, 2001; World Bank, 2012; Yamada et al., 2015). These previous studies referred to overall low achievement in these exams while highlighting the disparity between opportunities in Malé and those in the atolls. While enrollment figures for secondary education are trending up (Figure 9), there are still many Maldivian students for whom the progression from lower secondary education examinations (O level) to completion of higher secondary examinations (A level) remains a real challenge (Figure 10).

Our analysis considered trends in enrollment and achievement levels from 2005. The O-level examination results reveal an upward trajectory in national O-level pass rates (Figure 4) and a closing of the disparity between Malé and some atoll schools (Figure 5). In fact, Figure 7 shows that some atolls outperformed Malé in terms of achievement. The ESA (MoE, 2019) also refers to this rise in pass rates, noting education policy shifts. In this respect we refer to two relevant policies. First, the MoE implemented a specific policy to promote a rise in O-level attainment levels (MoE, 2010). Like the ESA (MoE, 2019), we also found that fewer students are sitting the GCE O-level examinations. Therefore, this rise in O-level pass rates should, according to the ESA, be “viewed with caution” (p. 36). Secondly, we note the availability of school-based TVET programs (Table 1 and Figure 8) that offer alternatives for students, recognizing that the O-level program does not suit all students. The ESA (MoE, 2019, p. 87) reports that “students who were not performing well academically were channeled to alternative, vocationally/technically oriented pathways and did not sit the GCE O/L exams”. Channeling students into TVET programs assists the schools’ O-level completion rates and could explain the leveling of the drop in O-level completion numbers in 2014 shown in Figure 10.

Concerns were raised in the ESA (MoE, 2019) about the Dhasvaaru program. Parents developed a perception that the programs were forced upon students who were labeled as incapable of succeeding in their external examinations (MoE, 2019). The drop in numbers in 2018 for both the TVET programs (Table 1; Figure 8) may in part be explained by the stigma attached to the program (Hussain, 2020). In the Education Sector Plan 2019–2023 (MoE and MoHE, 2019), the Ministry of Education expressed a need to reassess the Dhasvaaru TVET program. Consequently, a new TVET program, under a new banner called Heyvalla, was officially announced in the Maldivian media in 2020 (Hussain, 2020) to be piloted at several schools. However, no official policy document has yet been released. As indicated in the media report, the former Dhasvaaru program targeted low-performing students, but did not provide students with adequate literacy and numeracy skills for employment. Instead the Heyvalla program is to be made available to any student who has completed Grade 10. Our analysis finds that the TVET program may have had a stabilizing effect in the previously downward trend in O-level completion rates amongst students based in the atolls, yet a negative perception of the Dhasvaaru program has grown.

Given the high profile afforded to O-level outcomes in the Maldives, out-of-school private tuition to promote exam success has continued to grow. The ESA (MoE, 2019) highlighted the need to understand the motivations of the large numbers of students seeking tuition outside of school instruction. Mariya (2016), specifically investigating private tuition in the Maldives, found that parents made sacrifices to pay for tuition as they perceived that without this additional support their child’s progress would be jeopardized. Mariya (2016) also found that most teachers, across her two research sites, provided private tuition to students. These points support Yamada et al.’s (2015) findings that the extra support provided by families to support students to prepare for exams was a major influencing factor in exam success.

This discussion so far raises questions about the perceived adequacy of school-based instruction in preparing students for examinations. Tuition typically utilizes the strategy of completing past papers, emphasizing short-term success rather than long-term learning and conceptual understanding. Furthermore, Mariya (2016, p. 125) argues that the reliance on O-level results meant that “learning was passive in an examination-oriented, teacher-dominated mode”. Relying on O-level results as the measure of success prevented teachers from employing more learner-centered strategies, for fear of losing teaching time and out of concerns that noisy classrooms would be frowned upon. Whilst the NCF was meant to provide flexibility to teachers, the O-level results can be seen to deter teachers from expanding their range of teaching methods consistent with the pedagogical vision of the NCF. The conflicting messages are articulated by Barrow and Leu (2006):

Teachers juggle the demands of a misaligned system. When teachers reach their classrooms, they often face contradictions. The crowded and rigid curriculum and textbooks, filled with information that must be memorized for examinations…raises questions about how teachers should practice in the midst of such an apparent misalignment. (p. 6)

Whilst Mariya’s study relates to the period prior to the implementation of the NCF, similar challenges continue to be raised. For example, the MoE (2019) refers to the need to improve higher order thinking skills, and to train teachers in new curricula to promote high level cognitive skills such as inquiry methods. This follows a report in 2014 which found that students did not perform well on questions requiring conceptual understanding (UNDP, 2014). Yamada et al. (2015) pointed to a disjuncture between the pedagogical vision of the NCF and what O-level examinations measure. They contend that the exam-driven system promotes transmission teaching methods rather than the NCF’s vision for learner-centered pedagogy, arguing the reform should increase “ties between the curriculum, exams, classroom teaching, and teacher training in a more comprehensive manner” (p. 68). A further point they make is that the context of exams is at odds with the lives of Maldivian students. Hence the focus on O-level results actually detracts from the pedagogical vision embedded within the NCF to make learning relevant and meaningful for students.

The A-level trends remain challenging, with a notable attrition between O- and A-level enrollment and achievement (Figure 9, Figure 10). Success in A-level examinations remains problematic globally, as Stromquist (2020) notes. As A level is offered only on larger islands, some students may have to leave their island and family for the opportunity to complete higher secondary schooling. Further, the pass rate remains low, proving that many students are not achieving success at this level. This has implications for young people’s ability to engage with higher education opportunities (MoE, 2019). Considering the president’s statement regarding the need for young people to play a critical role in building back following the Covid-19 pandemic, these ongoing results of poor performance in HSE raise additional pressures.

Additionally, the comparatively low HSE completion, particularly in the atolls (Figure 10), may have critical implications for national development. The atolls’ lower levels of secondary education completion in comparison to the capital may be a key factor in the fewer years of schooling (YoS) and high levels of multidimensional poverty (MPI) in the atolls. These figures underline the high negative impact that fewer years of schooling can have on the socioeconomic development of Maldivian populations for the whole nation, particularly for the atolls. Further research that establishes the relationship between atoll poverty levels and YoS at secondary education for the five underperforming atolls (Raa, Alif Dhaal, Thaa, Laamu, and Gnaviyani) would provide additional insights into overcoming barriers.

Our analysis also highlights that the Maldives demonstrates a contrasting picture of HSE gender participation and achievement to the South Asian region (UNICEF, 2021). Latest available UNICEF data shows that while in the South Asian region, girls’ completion in HSE has been about 5 percentage points lower than boys, female HSE completion rates in the Maldives are 10–20% above that of males. There may be lower YoS for males given that, since 2013, males’ engagement in HSE has been lower than females, with a net enrollment ratio consistently below 40% (Figure 9). Further, boys performed considerably lower than girls, especially in the atolls (Figure 6). This has a subsequent effect on tertiary education, where females have a notably higher level of engagement than males: 60% and 17% gross enrollment ratios respectively (UNESCO, 2021). Despite higher educational engagement and outcomes for females, their engagement in the Maldivian employment sectors is surprisingly low. According to the most recently available data, males’ labor force participation rate is 75%, while for females it is 42% (National Bureau of Statistics, 2016). These findings indicate a need to investigate the factors impacting boys’ educational achievement in secondary education and girls’ higher educational outcomes yet lower contribution to employment activity in the Maldives.

With the exams enduring over time, Bray and Adam (2001, p. 234) make an important point: “At the time Maldives embarked on the link with the London-based examination board, the strategy seemed very appropriate. Numbers of candidates were small, the country had practically no domestic expertise to operate examinations of this type”. These circumstances have since changed. The introduction of O- and A-level examinations was a product of the needs of an expanding education system in English-medium Malé schools in the 1980s. As Yamada et al. (2015) conclude:

…the outcome shows bias to the elites—to those with better socioeconomic background and better family support for education. This elitist bias is different in nature from the ones pointed out in the literature on the cases where access to the O-level system itself was limited to the elites. In Maldives, the students who attend classes in ordinary public secondary schools do not have very high chances of getting diplomas unless they have support for study at home. (p. 67)

With the ongoing disparity between Malé and many atolls demonstrated in our data, we can argue that the impact of the current examination system serves as a process of selection rather than inclusion, given the number of students who do not participate and the number who are able to achieve success, particularly in HSE. This raises larger questions about who benefits and who does not.

Such challenges were noted by Bray and Adam (2001) 20 years ago. They outlined the concerns over the existing examination arrangements: cost, curriculum, bias in questions, elitism of the examinations, relevance to Maldivian daily life, and the backwash effect in the English medium of instruction (as instruction could not be in the mother tongue). Whilst the Maldivian authorities at the time had concerns, they chose to supplement the arrangements with Dhivehi and Islamic study national examinations rather than replace the whole system. Now many of the same challenges remain.

Bray and Adam (2001) presented some alternatives to the British examinations, referring to the needs of small states. These alternatives included options such as (a) national examinations like those in Fiji and Nauru, (b) regional examinations where states group together, and (c) metropolitan examinations where small states are clients of examination boards in larger countries. Such options would offer the opportunity to localize and contextualize the secondary curriculum to needs of a small island state like the Maldives. Such reconsideration may achieve a more equitable examination system that addresses regional equalities within the country, especially in the achievement of A level in HSE, as discussed in our findings. In fact, such an attempt was first made in the 1980s, when local education authorities collaborated with Cambridge’s International Examinations in Fisheries Science. The syllabus was produced locally, and the examinations were designed in liaison with Maldivian authorities by UK-based examination boards (Hameed & Moosa, 1986; Yamada et al., 2015). This indicates potential scope to extend such efforts to the wider curriculum.

Another option can be found in the Pacific, where there is currently an examination board (Educational Quality and Assessment Program) overseeing the South Pacific Form Seven Certificate (EQAP, 2021). This supports regional and national education and may present a model that could support small states within the Indian Ocean who seek a certificate that is more contextualized to the needs of Maldives students.

Preferences toward these international examinations have already been identified. As Bray and Adam (2001) report, the British examinations were perceived to offer external credibility. Further to this, Yamada et al. (2015) assert, parents prefer international certificates as they provide options to study abroad. Such perceptions are important and have helped cement the British examinations as a pillar within the education system. However, the evidence indicates the current examinations clearly benefit some over others. The need for Maldivian students to study abroad has lessened substantially with the establishment of two national universities and multiple private providers of higher education options, many with flexible and alternative delivery for students in the islands. The options presented by Bray and Adam (2001) provide a good starting point to think beyond the reliance on the British O- and A-level examinations in the Maldives.

## Conclusion

The impacts of the Covid-19 pandemic and the disruption to the status quo of schooling, whether through extended school lockdowns or disrupted assessment regimes, present an opportunity to reflect on what best suits the needs of Maldivian students. Our analysis indicates that the current secondary education system and examination regime in the Maldives continue to benefit some students over others. Its existence, and the notions of it being merit-based and fair, run counter to the outcomes we have illustrated, with disparity across atolls and success remaining out of reach for many students. While there has been some improvement in student achievement in the GCE O level in lower secondary education (LSE) in some atolls, success in secondary education remains unattainable for many. Moreover, achievement for A level in higher secondary education (HSE) shows only modest improvements. Investigating the reasons for this and the potential explanatory factors, such as atoll-level poverty and social inequity, is beyond the scope of this study but is worthy of further investigation.

Returning to our starting point that “We cannot do away with exams—parents believe in them, so does the wider community” (UNESCO, 2020a, para. 6), we turn to Sweeney (2020) in an analysis of the Covid-19 circumstances. He argues that if there is any hope of addressing the structural issues that disadvantage some students, we need to “end the fixation on A-level exams as the ‘gold standard’” (para. 14). The 2030 Education Agenda to “build systems that are inclusive, equitable, and relevant to all learners” (UNESCO, 2020b, para. 4) presents a call for action. Understanding the strengths and weaknesses of the current circumstances is the first step in considering options for the future, both for the Maldives and other countries relying on this examination regime.
